# Novel *Yersinia enterocolitica* Prophages and a Comparative Analysis of Genomic Diversity

**DOI:** 10.3389/fmicb.2019.01184

**Published:** 2019-05-29

**Authors:** Junrong Liang, Zengqiang Kou, Shuai Qin, Yuhuang Chen, Zhenpeng Li, Chuchu Li, Ran Duan, Huijing Hao, Tao Zha, Wenpeng Gu, Yuanming Huang, Meng Xiao, Huaiqi Jing, Xin Wang

**Affiliations:** ^1^State Key Laboratory of Infectious Disease Prevention and Control, Collaborative Innovation Center for Diagnosis and Treatment of Infectious Diseases – Chinese Center for Disease Control and Prevention, National Institute for Communicable Disease Control and Prevention, Beijing, China; ^2^Shandong Provincial Centre for Disease Control and Prevention, Jinan, China; ^3^Shenzhen Nanshan Maternity and Child Heath Care Hospital, Shenzhen, China; ^4^Department of Pathogenic Biology, School of Medical Science, Jiangsu University, Zhenjiang, China; ^5^Chang Ping Women and Children Health Care Hospital, Beijing, China; ^6^Wuhu Municipal Centre for Disease Control and Prevention, Wuhu, China; ^7^Yunnan Provincial Centre for Disease Control and Prevention, Kunming, China

**Keywords:** *Yersinia enterocolitica*, prophage, *Podoviridae*, *Myoviridae*, comparative genomic analysis

## Abstract

*Yersinia enterocolitica* is a major agent of foodborne diseases worldwide. Prophage plays an important role in the genetic evolution of the bacterial genome. Little is known about the genetic information about prophages in the genome of *Y. enterocolitica*, and no pathogenic *Y. enterocolitica* prophages have been described. In this study, we induced and described the genomes of six prophages from pathogenic *Y. enterocolitica* for the first time. Phylogenetic analysis based on whole genome sequencing revealed that these novel *Yersinia* phages are genetically distinct from the previously reported phages, showing considerable genetic diversity. Interestingly, the prophages induced from O:3 and O:9 *Y. enterocolitica* showed different genomic sequences and morphology but highly conserved among the same serotype strains, which classified into two diverse clusters. The three long-tailed *Myoviridae* prophages induced from serotype O:3 *Y. enterocolitica* were highly conserved, shared ≥99.99% identity and forming genotypic cluster A; the three *Podoviridae* prophages induced from the serotype O:9 strains formed cluster B, also shared more than 99.90% identity with one another. Cluster A was most closely related to O:5 non-pathogenic *Y. enterocolitica* prophage PY54 (61.72% identity). The genetic polymorphism of these two kinds of prophages and highly conserved among the same serotype strains, suggested a possible shared evolutionary past for these phages: originated from distinct ancestors, and entered pathogenic *Y. enterocolitica* as extrachromosomal genetic components during evolution when facing selective pressure. These results are critically important for further understanding of phage roles in host physiology and the pathology of disease.

## Introduction

As the most abundant microorganisms on the planet (Brussow and Hendrix, 2002; Olszak et al., 2017), phages can be divided into two kinds. The lytic phages contain a copy of the phage genome packaged in its capsid, which is built into its quaternary structure prior to lysing the host cell and its subsequent release. In contrast, the temperate phages integrate into bacterial genomes as prophages and represent an important source of genetic variation on bacterial evolution, such as producing toxins responsible for the virulence of major pathogens, producing cell adhesion molecules, nutrient uptake, immune response evasion, fimbriae, and others (Ikeda and Tomizawa, 1968; Briani et al., 2001; Chen and Lu, 2002; Abedon and Lejeune, 2007; St-Pierre and Endy, 2008; Ariff et al., 2015). These prophage-bacterial combinations are described as lysogenic bacteria or lysogens. With the development of high-throughput sequencing technology, many genomes have been sequenced, and numerous prophages have been identified. These results can further our understanding of roles in phage host physiology and the pathology of disease.

*Yersinia enterocolitica* is an important zoonotic pathogen leading to human and animal enteric infections and includes three lineages: avirulent strains belonging to biotype 1A, highly pathogenic strains of biotype 1B, and weakly pathogenic strains of biotypes 2–5 (Cornelis et al., 1998; Revell and Miller, 2001; Reuter et al., 2014; McNally et al., 2016). Compared with other countries, there is no pathogenic O:8 strains in China, only serotypes O:3 and O:9 *Y. enterocolitica* strains, which carry virulence determinants that can cause human infections (Wang et al., 2008, 2009). In many years of *Y. enterocolitica* surveillance in China, we found that the O:3 serotype strains have replaced O:9 strains and become the main pathogenic *Y. enterocolitica* (Wang et al., 2009; Liang et al., 2015). Currently, there are 15 *Y. enterocolitica* and 27 *Yersinia* phages of full-length genomic sequences in the National Center for Biotechnology Information (NCBI) database. Genomic sequencing of *Y. enterocolitica* has made it possible to identify prophages and perform comparative genomic analysis of phage sequences. To the best of our knowledge, several O:3 and O:9 *Yersinia* lytic bacteriophages have been described and some can subtype *Y. enterocolitica* (Leon-Velarde et al., 2016; Jun et al., 2018; Salem and Skurnik, 2018). The lytic phages phiYe-F10 and φYeO3-12 displayed specificity for *Y. enterocolitica O:3* (Pajunen et al., 2003; Liang et al., 2016), phage φR1-37 has a broader host range within *Y. enterocolitica* also it can infect some *Y. intermedia* and *Y. similis* strains (Kiljunen et al., 2005), and phage vB_YenP_AP5 can form plaques only on *Y. enterocolitica* serotypes O:3, O:*2*, and *O:1* (Leon-Velarde et al., 2014). However, there have not been any reports describing the prophages of main pathogenic O:3 and O:9 *Y. enterocolitica*, and only one *Y. enterocolitica* prophage has been isolated. The temperate phage PY54 isolated from non-pathogenic *Y. enterocolitica* O:5 strain has a lambda-like morphology and infects the O:5 strains and pathogenic O:5,27 strains (Hertwig et al., 2003a,b). In this study, we first induced 6 novel prophages from serotype O:3 and O:9 pathogenic *Y. enterocolitica* and performed comparative genomic and phylogenetic analyses with other phages.

## Materials and Methods

### Bacterial Strains and Induction of Prophages

The lysogenic phages were induced using mitomycin C (Ivánovics et al., 1976; Faelen et al., 1993). Strains were grown overnight on brain heart infusion [BHI] Agar (Oxoid) at 27°C. The colonies were transferred to BHI culture medium and shaken to OD_600_ = 0.4∼0.8 at 27°C, followed by four additions to BHI culture medium for further replication. The experimental groups were induced with 0.5 μg/ml mitomycin C (Sigma) and the control group received nothing and was incubated for 8–14 h at 27°C with gentle shaking. If the difference of OD_600_ between the experimental group and control group was equal or greater than 0.5, it was suggested that bacteriophage particles may be induced. We confirmed the prophages with a double-layer plaque assay at 25°C. The supernatant was filtered through a sterile disposable filter of 0.45 μm pore size (Thermo Fisher Scientific, Mississauga, ON, Canada) and standard double agar overlay plaque assays were used to identify plaques.

### Electron Microscopy

The presence of induced phages were confirmed by transmission electron microscopy. Filtered phage lysates were pelleted at 25,000 ×*g* for 1 h at 4°C, using a Beckman high-speed centrifuge and a JA-18.1 fixed-angle rotor (Beckman, Palo Alto, CA, United States). The phage pellets were washed twice under the same conditions in neutral 0.1 M ammonium acetate. The final phage sediment was re-suspended in 150 μL of SM-buffer supplemented with 5 mM CaCl_2_. Samples were then deposited onto carbon-coated Formvar films on copper grids and stained with 20 μl of 2% potassium phosphotungstate (PT, pH 7.2). The dye was removed with filter paper and the sample was air dried and examined under a Tecnai G2 F20 transmission electron microscope (FEI, Hillsboro, OR, United States), operating at 120 keV. Images were collected and analyzed using Digital Micrograph^TM^ Software (Gatan, Pleasanton, CA, United States).

### Isolation of Phage DNA

To separate the phage, the phage inducing lysate was centrifuged at 10,000 ×*g* for 15 min at 4°C and the supernatant was filtered through a 0.22 μm low protein binding filter (Millipore, United States). Contaminating nucleic acids in the supernatant were digested with pancreatic DNase I and RNase A, and each was added to obtain a final concentration of 10 μg/mL (Sigma-Aldrich Canada Ltd., Oakville, ON, United States) and incubated for 15 min at 37°C. DNA isolation was then performed according to Molecular Cloning: A Laboratory Manual, Third Edition (Sambrook J., Russell D.W. Molecular Cloning: A Laboratory Manual. 3rd ed. Volme 1 Cold Spring Harbor Press; Cold Spring Harbor, NY, United States: 2001), with minor modifications. For phage DNA purification the suspension was extracted twice with an equal volume of phenol-chloroform, once with chloroform and precipitated with ethanol. The pellets were washed in 70% ethanol, vacuum dried, and resuspended in 20 ml distilled water. PCR verification was performed on 1 ml of the phage DNA preparation.

### Genome Sequencing and Assembly

We tested the quality of the whole genomes of the phages with Qubit3.0 (Life Technology, United States) followed by NEB Next Ultra DNA Library Prep Kit for library construction. Whole genome sequencing was carried out using the Illumina HiSeq2500 (PE25) genome analyser. Generated reads were assembled using SPAdes. The assembled contigs were annotated using Rapid Annotation using Subsystem Technology (RAST, version 2.0) and PHASTER web server (PHAge Search Tool Enhanced Release^1^) (Fancello et al., 2011; Arndt et al., 2016, 2017).

### Prophage Sequence Detection

The complete genomes of *Y. enterocolitica* were downloaded from NCBI database, which were analyzed by PHASTER to identify the presence of prophages. The prophages were identified as intact, questionable or incomplete by PHASTER. Intact phages were estimated as complete functional phages, and functionality needs to be tested with plaque formation analyses. Questionable and incomplete phages do not contain sufficient prophage genes considered as unfunctional phages (Arndt et al., 2017).

We analyzed the strains of O:9 serotype *Y. enterocolitica* with the designed O:9 serotype phage-specific primers (F: TCAGGTAGTCTGACTTGACCGA, R: TCACGCTGGATGTGCCTATTGT).

### Comparative Genomic Analyses

Data from 21 available whole *Yersinia* (pro)phages genomic sequences and 85 of the other *Enterobacteriaceae* phages were downloaded from the NCBI website and analyzed with the 6 *Yersinia enterocolitica* prophage sequences in this study (Supplementary Table S2). The comparison heat map of the phage genome structure was drawn as a flow diagram: first the non-redundant genes were obtained for all the genomes using cd-hit; second, an m^∗^n matrix (m indicated the number of pangenes, n indicated the number of phages) was constructed. If the individual gene existed on the specific phage, the corresponding position of the matrix was tagged as 1; otherwise, it was tagged as 0. Euclidean distance was computed between the different phages based on the matrix, and a new matrix n^∗^n was constructed. The item of the matrix indicated the distance of the corresponding phage. Last, the heat map was constructed using the pheatmap package. Average nucleotide identity (ANI) of the phage genomes were calculated using the BLASTn algorithm in JSpecies 1.2.1 (Richter and Rossello-Mora, 2009).

Comparative genomic analyses were carried out using the BLAST software with an *e*-value of 1e-2, and the alignments of >1 kb were retained. PRODIGIAL v2.6 was used to predict the CDS for all of the phage genomes. A Perl script was used to draw the phage genome structure.

## Results

### Induction of Lysogenic Bacteriophages and Electron Micrographs of Phage Particles

**FIGURE 1:**
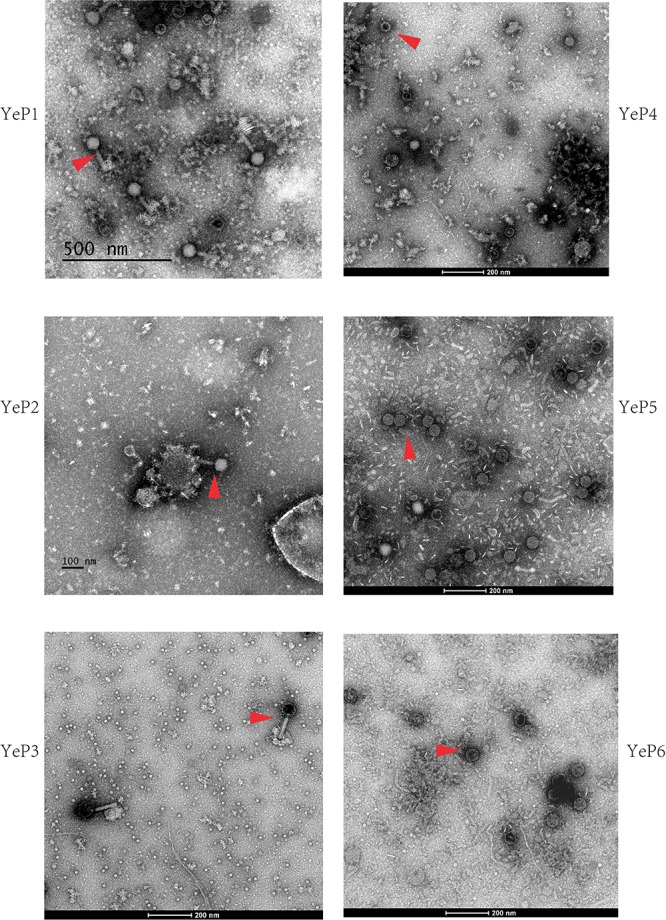
The electron micrographs of the six prophages. The phages are negatively stained with 2% potassium phosphotungstate and showed with red arrows. Scale bar indicates size in nm.

Altogether, 6 bacteriophages were induced from *Y. enterocolitica* with mitomycin C (Table 1). The induced prophages are able to form turbid plaques on *Y. enterocolitica* isolates that do not harbor the cognate prophage, but they do not infect the *Yersinia pestis*, *Yersinia pseudotuberculosis*, *Escherichia coli*, and *Salmonella* isolates we tested. The bacteriophages were negatively stained and examined using transmission electron microscopy. These phages showed hexagonal outlines, indicating their icosahedral nature. However, approximately 95% of the O:3 phage particles released and directly analyzed in the crude lysate were defective, consisting of DNA-filled heads without tails. The phages YeP1, YeP2 and YeP3 were induced from the 3/O:3 *Y. enterocolitica* serotype and were characterized by icosahedral symmetry head (ranged from 56.0 nm to 64.0 nm) and long (ca. 140–160 nm), flexible, contractile tails which belonged to *Myoviridae* family. YeP4, YeP5, and YeP6 were induced from the O:9 *Y. enterocolitica* serotype, with icosahedral head (ranged from 52.0 nm to 58.0 nm) and very short non-contractile tail, and the morphological group corresponds to the *Podoviridae* family (Figure 1).

**Table 1:** The information of lysogenic bacteria from which *Y. enterocolitica* temperate bacteriophages were induced.

Phage	Size (kb)	GC %	Lysogenic bacteria	Biosero type	Source	Region	Year	*ail*	*ystA*	*ystB*	*yadA*	*virF*	*rfbc*
YeP1	39.5	48.1	BJ2014-1008329276	3/O:3	Patient feces	Beijing	2014	+	+	-	+	+	+
YeP2	39.8	48.0	BJ2014-1008297168	3/O:3	Patient feces	Beijing	2014	+	+	-	+	+	+
YeP3	39.8	48.0	QH2012-y91	3/O:3	Pig tonsils	Qinghai	2012	+	+	-	+	+	+
YeP4	35.0	50.4	LN1996-YE105.5R	3/O:9	Patient feces	Liaoning	1996	+	+	-	+	+	-
													
YeP5	35.0	50.1	JL2002-6	2/O:9	Mouse feces	Jilin	2002	+	+	-	+	+	-
YeP6	35.0	50.1	JL2004-21	2/O:9	Mouse feces	Jilin	2004	+	+	-	+	+	-
Phage PY54^∗^	46.3	44.6	*Y. e* strain 29854	1A/O:5	Food	Germany	2003	Unknown					


*^∗^PY54 (NC_005069.1) isolated from a non-pathogenic *Y. enterocolitica* O:5 strain has a lambda-like morphology and infects O:5 strains and pathogenic O:5,27 strains. The phage genome comprises double-stranded DNA and contains 3′- protruding ends of 10 nucleotides.*

### Prophages Genome Analysis

**FIGURE 2:**
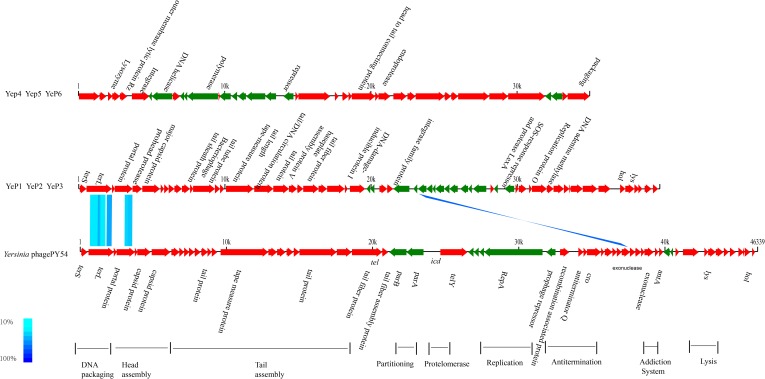
Genomic comparison between prophages from O:3/O:9 *Yersinia enterocolitica* strain and Phage PY54. Each arrow identifies an open reading frame. Color shading was used to show the amino acid identity between the two ORFs. The absence of shading indicates no significant similarity. The phage genome structure were classified into two groups: prophage YeP1, YeP2 and YeP3 were from O:3 *Y. enterocolitica*; and YeP4, YeP5 and YeP6 were from O:9 Y. enterocolitica. The genome sequence of *Y. enterocolitica* bacteriophage PY54 was from GenBank (Accession number NC_005069).

The phages that resulted from induction of the prophages in the lysogens were purified and their DNA was sequenced. Altogether, 1.5G raw data was available for each phage with a sequencing depth of >8000×. The cleaned raw data obtained had Q30 >80% after quality checks for sequencing. We assembled the raw data using SPAdes genome assembler version 3.10.1. Each of the phage genomes was assembled as a circular molecule when the sequencing was completed. The phage genome sizes ranged from 34,969 bp to 39,751 bp, and the G+C contents varied between 48.0 and 50.4% (Table 1), similar to the 48.5 ± 1.5% reported for their *Y. enterocolitica* hosts. Phage YeP1 induced from strain BJ20141008329276 had a total genome length of 39,492 bp; YeP2 induced from BJ20141008297168 had a total genome size of 39,751 bp; YeP3 induced from strain QH2012-Y91 with 39,751 bp. Features of the open reading frames (ORFs) of each prophage were listed on Supplementary Table S1. YeP1 phage group from serotype O:3 strains with 61 proposed ORFs and three tRNA were predicted, and predicted functions were attributed to 38 of the ORFs based on similarities of the predicted products to the known proteins. Phage YeP4 induced from strain YE105.5R had a genome length of 35,029 bp; YeP5 and YeP6 induced from strain JL2002-6 and JL2004-21 had genome length of 34,969 bp, both with 39 ORFS predicted, of which 16 had predicted functions. The genomic structure of O:3 and O:9 *Y. enterocolitica* prophages were shown as Figure 2. PY54 was isolated from a non-pathogenic *Y. enterocolitica* O:5 strain and was the first temperate *Yersinia* phage that had been sequenced and characterized by Hertwig et al. (2003a,b).

### Comparative Genomic Analyses

**FIGURE 3:**
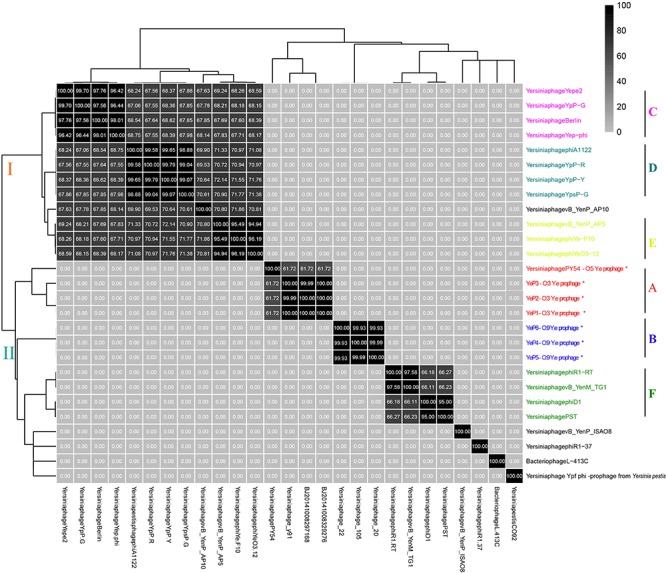
The sequence average nucleotide identity of phage genomes. ^∗^Indicates the temperate phage of *Yersinia*.

**FIGURE 4:**
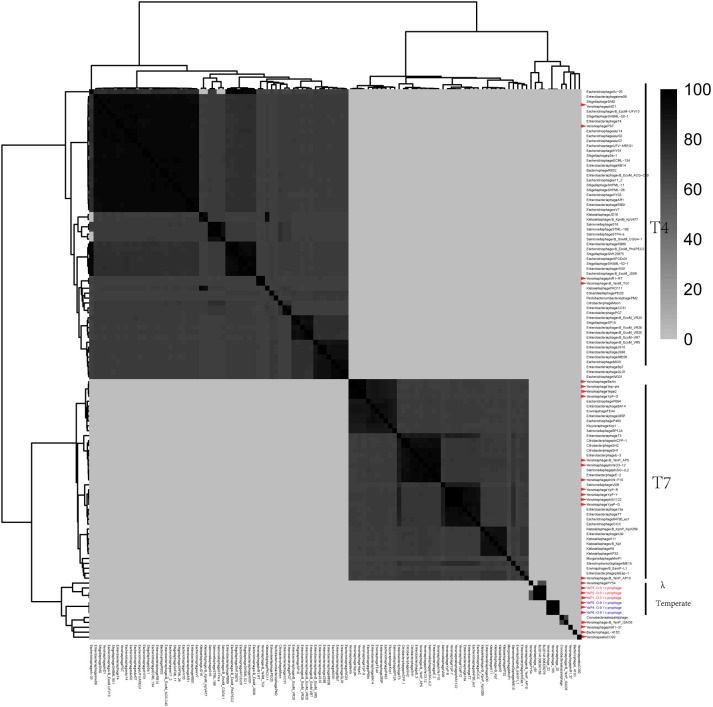
The comparative genomic structure analyses of all *Yersinia* phages and some of the *Enterobacteriaceae* phages.

The six *Y. enterocolitica* prophage genome sequences showed little similarities with the previously reported phages (Figure 3, 4 and Supplementary Figure S1). Phages YeP1, YeP2, and YeP3 induced from the O:3 *Y. enterocolitica* serotype clustered together with an identity of more than 99.99%; the three O:9 *Y. enterocolitica* serotype lysogenic phages (YeP4, YeP5, and YeP6) were identity up to 99.93% (Figure 3). Little similarity existed between the lysogenic phages from O:3 and those from the O:9 serotype of *Y. enterocolitica* (Figure 2, 3). The comparative genomic analyses showed that the *Yersinia* phages were sorted into five large clusters (cluster A, cluster B, cluster C, cluster D, and cluster E) and 10 single clusters based on genomic similarities (Figure 3 and Supplementary Figure S1). Cluster A contained three O:3 prophages: YeP1, YeP2, and YeP3; cluster B contained three O:9 lysogenic phages YeP4, YeP5 and YeP6; and cluster C contained three O:3 serotype-specific virulent phages: phiYeO3-12, *Yersinia* phage vB_YenP_AP5 and *Yersinia* Phage phiYe-F10. *Y. enterocolitica* lysogenic phage PY54 showed 61.72% similarity to the O:3 *Y. enterocolitica* lysogenic phages (Figure 3). However, the *Y. enterocolitica* lysogenic phage PY54 formed a separate cluster. *Y. pestis* phages were divided into two large clusters: cluster D contained YepΦ, Berlin, Yepe2, and YpP-G, cluster E contained YpP-Y, YpP-R, YpsP-G, and ΦA1122, and the rest were all sepereted (Figure 4). The phage genome sequence average nucleotide identity showed the similarity results (Figure 3).

The closely related phage of O:3 *Y. enterocolitica* prophage was prophage PY54 (ANI: 61.72%) and shared the same new branch in the phylogenetic tree (Figure 3 and Supplementary Figure S2). We identified homology with only very short protein fragments of *Yersinia* phages PY54 compared to YeP2 prophage, and mauve alignment showed 1,326 SNPs between them. The peg.2 (533–2230 bp) of YeP2 was similar to the Phage terminase of PY54 (PY54p02, protein_id: NP_892047.1), with identity 63%; the peg.3 (23208–23787 bp) of YeP2 was similar to portal protein of PY54 (PY54p03, protein_id: NP_892048.1) with identity 71%; and the peg.33 (23229–23780 bp) of YeP2 was similar to exonuclease (PY54p50, protein_id: NP_892096.1) with identity 82%. The proteins encoded by the remaining ORFs had no homology with other known phages proteins.

### Identified the Prophage-Like Elements of *Yersinia enterocolitica*

The prophages were found among all of the 15 genomes of *Y. enterocolitica* from GenBank with PHASTER. Altogether, 117 prophage-like elements were identified with 55 intact prophages and 62 defective prophages. Their size ranged from 6 kb to 102.9 kb. The average of prophages per genome for *Y. enterocolitica* was 7.8.

Six prophage-like elements were identified within the whole genomes of *Yersinia enterocolitica* subsp. *palearctica* 105.5R(r) (CP002246.1) (Table 2). The genome sizes of prophages ranged from approximately 11.2–54.9 kb, and the GC content varied between 41.17 and 48.87%. Only two prophage regions were predicted intact with the region’s total scores 150 and 116, and the remaining four were incomplete or questionable. The lysogenic bacteriophage YeP4 was induced from YE105.5R genome (CP002246.1), the coordinates was located from 1584464 to 1619508, which determined by BLASTN with sequences alignment. The sequence of YeP4 (from 3500 to 35029) was highly homologous to YE105.5R genome (from 1584464 to 1619508) with nucleotide identities higher than 99.99%. And the sequence of YeP4 (from 1 to 3518) was identical to YE105.5R genome (from 1615991 to 1619508).

**Table 2:** *Yersinia enterocolitica* subsp. palearctica 105.5R(r) genomic prophage prediction results.

Strain	Size (Kb)	GC%	Region length (Kb)	Com	Score	Total proteins	Region position	Most common PHAGE (NCBI No.)	GC %
*Yersinia enterocolitica subsp. palearctica* 105.5R(r)	45.52	47.00	30.0	I	50	17	1411309–1441385	PHAGE_Acanth_mimivirus (NA)	48.00
			54.9	I	60	33	1569397–1624380^∗^	APSE-2 (NC_011551)	48.87
			31.0	T	150	44	2080617–2111707	Salmon_ RE_2010 (NC_019488)	47.23
			19.7	T	116	30	2476620–2496342	Entero_I2_2 (NC_00133)	41.17
			13.8	I	40	11	2720603–2734483	Entero_ PsP3 (NC_005340)	43.88
			11.2	I	40	11	3301918–3313203	Salmonella phage SSU5 (GCA_000899335.1)	42.42


*Com, completeness; I, incomplete; T, intact; NA, not available. The identified prophage-like sequences in *Yersinia enterocolitica* subsp. palearctica 105.5R(r) genomic with PHASTER web server. ^∗^The asterisk showed the region where the prophage YeP4 located (nucleotides 1584464 to 1619508) in the YE105.5R. The locations of the identify intact prophage were not where the prophage YeP2 induced. And the coordinate of the induced prophage existed between the identify incomplete phage region.*

### Phage Genome Sequences and Accession Numbers

The complete sequences of YeP1, YeP2, YeP3, YeP4, YeP5, and YeP6 were submitted to the NCBI databank under the Accession numbers as follows: MK733259, MK733260, MK733261, MK733262, MK733263, and MK733264, respectively. All of the sequencing data assembled were linear double strand DNA.

## Discussion

As we all know, prophages can play particularly important roles in bacterial evolution since phage-mediated mobility can result in faster gene evolution through increased rates of mutation, recombination, or adaptation (Casjens, 2003; Labrie et al., 2010). In addition, prophages also have an intimate association with the novel phenotypic properties of bacterial hosts, such as pathogenicity and genomic variation (Abedon and Lejeune, 2007; Fortier and Sekulovic, 2013; Pleska et al., 2018). Little is known about the genetic information of prophages of *Y. enterocolitica*, a major pathogen of human intestinal disease. In this study, we induced and isolated 6 prophages from *Y. enterocolitica*, which had few similarities compared to the genomic sequences of previously reported phages, suggesting that they were novel *Yersinia* phages and have considerable genetic diversity. Comparative genomic and phylogenetic analyses of prophage sequences revealed that the 27 *Yersinia* prophages could be sorted into 5 large clusters and 8 separate clusters. The three O:3 *Y. enterocolitica* prophages are from a common ancestor, with a hexagonal capsid, a long non-contractile tail and a sequence identity more than 99%, and as such, were denoted group A (Figure 3 and Supplementary Figure S1). The three *Podoviridae* O:9 *Y. enterocolitica* prophages, clustered as group B. However, there was no sequence similarities between group A and B, indicating that during the evolution of the O:3 and O:9 serotypes of *Y. enterocolitica*, they obtained different prophages that persistently presented in the genome as extrachromosomal genetic elements under selective pressure. In this study, the prophage sequence diversity in different *Y. enterocolitica* serovars, the findings presented suggest a possible different lysogenesis evolutionary past for these strains. *Salmonella enterica* prophage sequence profiles can reflected genome diversity and can be used for high discrimination subtyping (Mottawea et al., 2018). Prophages have also been used for bacterial typing in *E. coli*, *Streptococcus pneumoni*a, and *Bacillus anthracis* based on their ubiquitous and unique features (Sozhamannan et al., 2006; Romero et al., 2009; Kwon et al., 2013).

Prophage PY54 shared little nucleotide identity (61.72%) with prophage YeP1 and YeP2 (Figure 3) and could not be grouped into a cluster according to genomic similarity (Supplementary Figure S1). *Yersinia* phages and other T4 and T7 *Enterobacteriaceae* phages clustered together in comparative genomic analyses (Grose and Casjens, 2014; Figure 4), which implied that the *Y. enterocolitica* phage had a genetic relationship, to a certain extent, compared to other *Enterobacteriaceae* phages. The virulent phages of *Y. enterocolitica* O:3 serotypes, *Yersinia* phage phiYeO3-12, *Yersinia* phage phiYe-F10, and *Yersinia* phage vB_YenP_AP5, had a high genomic similarity and clustered together (Liang et al., 2016).

Among the 8 sequenced strains of *Y. pestis* virulent phages, Berlin, ΦA1122, Yepe2, YpP-G, Yep-phi, YpP-Y, YpP-R and YpsP-G all belonged to the T7 phage family, *Podoviridae* and *Caudovirales*, with the same size of the hexagonal structure head and a short non-stretching conical tail (Garcia et al., 2003; Schwudke et al., 2008; Zhao et al., 2011; Rashid et al., 2012). Although they are all double-stranded DNA viruses with the same genome size, alignments showed that the eight phages clustered into two groups: YepΦ group (YepΦ, Berlin, Yepe2, and YpP-G) and ΦA1122 group (YpP-Y, YpP-R, YpsP-G, and ΦA1122) (Figure 3). Interestingly, they exhibit a different host range (Filippov et al., 2012): ΦA1122 and YpP-G cannot infect some strains of *Y. pseudotuberculosis*, while YpP-Y, YpP-R, YpsP-G, and YpsP-PST can. In addition, YpP-Y, YpP-R, YpsP-G, YpsP-PST and ΦA1122 can also infect *E. coli* ATCC35401, *Klebsiella pneumoniae* env17, and *Shigella sonnei* S43-46 (probably as the common receptors for *Y. pestis* and *Shigella*) (Garcia et al., 2003). To date, the molecular interactions between bacteriophages and *Y. pestis* have been studied. The LPS and two OMPs (Ail and OmpF) are currently recognized as the receptor for bacteriophages of *Yersinia pestis* (Filippov et al., 2011; Kiljunen et al., 2011).

The genome of the lysogenic bacteria YE105.5R was completely sequenced by our laboratory (GenBank: CP002246.1) (Wang et al., 2011). The locations of the identify intact prophage were not where the prophage YeP2 induced. And the coordinate of the induced prophage existed between the identify incomplete phage region. The lysogenic bacteriophage YeP4 was induced from YE105.5R genome (CP002246.1), the coordinates was located from 1584464 to 1619508, which determined by BLASTN with sequences alignment. The sequence of YeP4 (from 3500 to 35029) was highly homologous to YE105.5R genome (from 1584464 to 1619508) with nucleotide identities higher than 99.99%. And the sequence of YeP4 (from 1 to 3518) was identical to YE105.5R genome (from 1615991 to 1619508). The YeP4 genome integrated into the YE105.5R chromosomal sites of YE105_RS07025 (protein_id = “WP_013649519.1”) and YE105_RS07220 (protein_id = “WP_013649558.1”). Blast screen of the NCBI database showed that, much to our surprise, YeP4 had similarities of up to 99% with sequences of *Y. enterocolitica* KNG22703 (GenBank: CP011286.1) (Range: 1374407–1403830), *Y. enterocolitica* 2516–2587 (GenBank: CP009838.1) (Range: 3262942–3292365) and *Y. enterocolitica* W22703 biovar 2, serovar O:9 (GenBank: FR718687.1) (Range: 1–29424). It is noteworthy that these strains were all O:9 serotypes, which indicated that prophage YeP4 can be found in the genomes of many O:9 serotype *Y. enterocolitica* strains from diverse localities. We analyzed 24 strains of O:9 serotype *Y. enterocolitica* with the designed O:9 serotype phage-specific primers, with 14 being positive. After that, we sequenced the genomes of two of these lysogenic bacteriophages, YeP5, and YeP6. The sequencing results showed that the identity of phage YeP5, YeP6, and YeP4 sequences was as high as 99.9%, implying a high similarity and prevalence of the O:9 serotype prophage. It is possible that, during the evolution of *Y. enterocolitica* O:9 serotype strains, they obtained the same phage due to selection pressure. However, YeP1 phage genomes shared low nucleotide identity with other available *Yersinia* genome sequences in GenBank.

In the 15 genomes analyzed 55 complete prophages were identified (data not show). YE105.5R contained 6 prophage regions, with 2 complete phages with intact genomes (2080617–2111707 and 2476620–2496342), and the remaining 4 prophages were incomplete or questionable (1411309–1441385, 1569397–1624380, 2720603–2734483, and 3301918–3313203). PHASTER web server results showed that prophage-like elements were identified in every complete *Y. enterocolitica* genomic sequence from the NCBI database. Interestingly, the induced lysogenic phage genome of YeP4 was located at 1584464–1619508 in YE105.5R. This position corresponds to an incomplete prophage region in the phaster prediction, with a score of 50 points; however, no prophages were induced in the two intact regions (scores 150 and 116) (Table 2). Thus, we concluded that the predicted regions of the prophages on phaster were inconsistent with the actual lysogenic phage regions, and the predicted intact phage regions were not actually the functional prophage regions.

The primary determinant in the infection of a bacterial host by a bacteriophage is the adsorption of the phage receptor binding proteins, e.g., the tail fiber, to the host receptor (Morita et al., 2002). Conversely, bacteriophages are capable of rapid adaptive responses to evolutionary changes in their hosts. As a defensive measure, evolution can modify the phage’s receptors binding proteins to achieve infection and kill the resistant bacterium. Its specificity depends on the complementarity between the phage receptor binding protein and the surface structure of the host bacterial receptor (Heilpern and Waldor, 2000). There may be a strong selection for fixing adaptive mutations, and tail fiber proteins can recognize a variety of host bacterial receptors (such as outer membrane proteins or lipopolysaccharide, LPS). Our previous studies have found that there is a correlation between tail fiber sequence and target serotype: the phage phiYe-F10, phiYeO3-12 and vB_YenP_AP5 display specificity for *Y. enterocolitica* O:3 were clustered together (Liang et al., 2016). The alignment showed high similarity among the prophage tail fiber proteins from the same serotype, which were consistent with earlier results. However, there are few similarities between different serotypes.

To the best of our knowledge, this is the first systematic analysis of temperate phages from pathogenic O:3 and O:9 *Y. enterocolitica*. The genomes and genetic information of *Y. enterocolitica* prophages play an important role in the analysis of the genetic evolution of the bacterial genome. This study showed that these novel genome of *Y. enterocolitica* prophages had diversity and highly conserved among the same serotype strains. The specificity of the phage tail fiber protein played a key role in the identification of surface receptors of different host bacteria. We suggested that the different tail fiber proteins from the O:3, O:9 serotype *Y. enterocolitica* lysogenic bacteriophages result in a difference in the specificity of the two phage hosts. These phages could also be used for specific and efficient detection of pathogenic *Y. enterocolitica*.

## Author Contributions

JL, ZK, ZL, and XW conceived and designed the experiments, sequenced, assembled, and annotated the genomes of prophages. XW and HJ supervised the study and contributed to manuscript writing. YC, WG, SQ, and YH performed the data analysis and assisted in the preparation of figures and text. CL and MX participated in the data interpretation. JL and ZL contributed to bioinformatic analysis. JL, RD, HH, TZ, and YC contributed to manuscript writing. All authors read and approved the final manuscript.

## Conflict of Interest Statement

The authors declare that the research was conducted in the absence of any commercial or financial relationships that could be construed as a potential conflict of interest.

## Footnotes

1 http://phaster.ca/

## Supplementary Material

FIGURE S1 Similarity matrix of 27 *Yersinia* phages and prophages based on the presence/absence of genes. The heatmap was generated based on the number of proteins shared by phages. Deeper shade of blue indicated a closer relationship.

FIGURE S2 The phylogenetic tree of the 27 *Yersinia* phages genome sequences using the program VICTOR (https://ggdc.dsmz.de/victor.php).

TABLE S1 Features of the open reading frames (ORFs) of six prophages.

TABLE S2 The (pro)phages used in this study.
