# Supplementary figures and images for: Novel Yersinia enterocolitica Prophages and a Comparative Analysis of Genomic Diversity
Source: Front Microbiol. 2019 May 29;10:1184. doi: 10.3389/fmicb.2019.01184 (PMC6548840; doi:10.3389/fmicb.2019.01184)

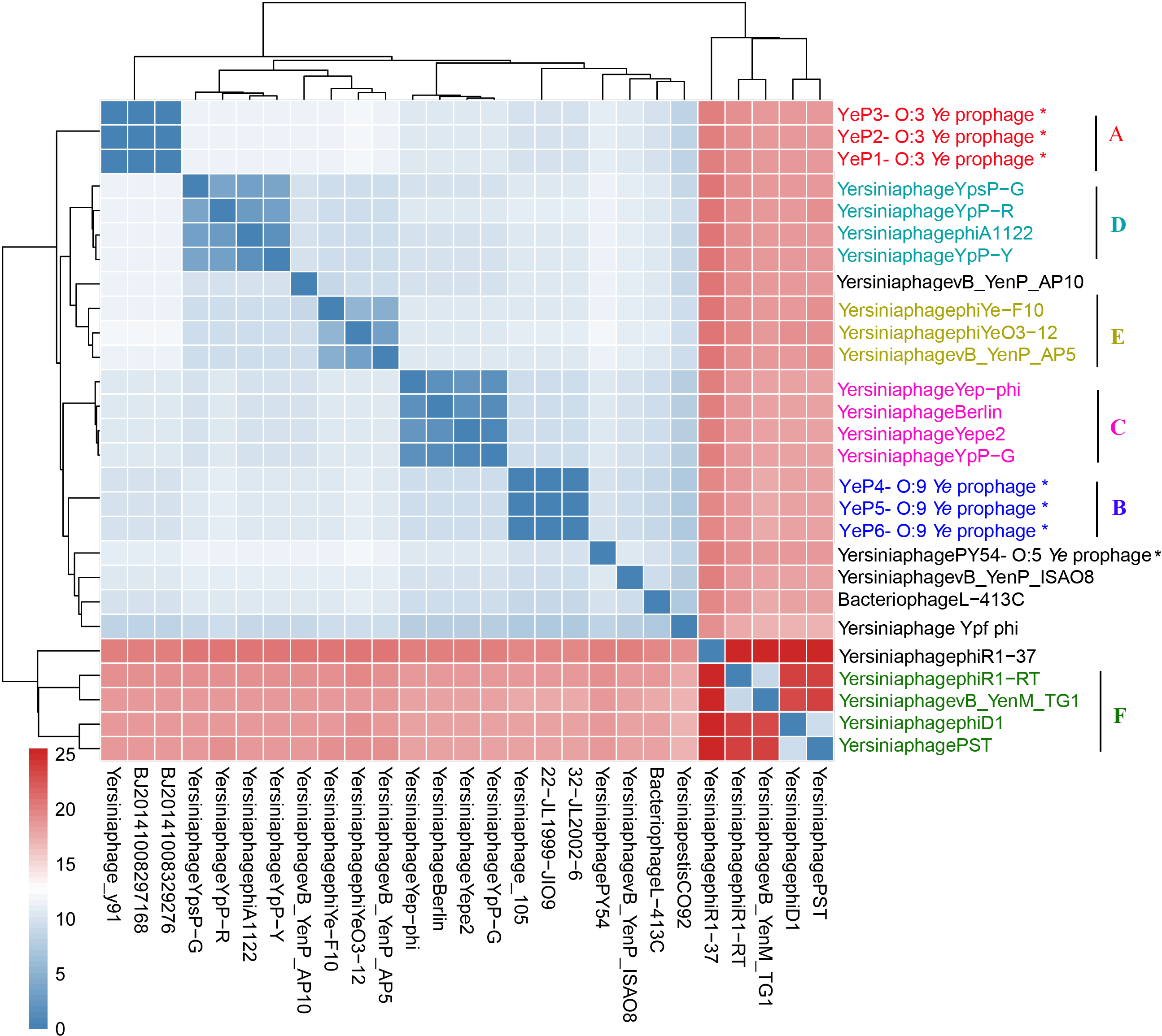

Supplement: FIGURE S1 — Similarity matrix of 27 Yersinia phages and prophages based on the presence/absence of genes. The heatmap was generated based on the number of proteins shared by phages. Deeper shade of blue indicated a closer relationship. [file Image_1.TIF]

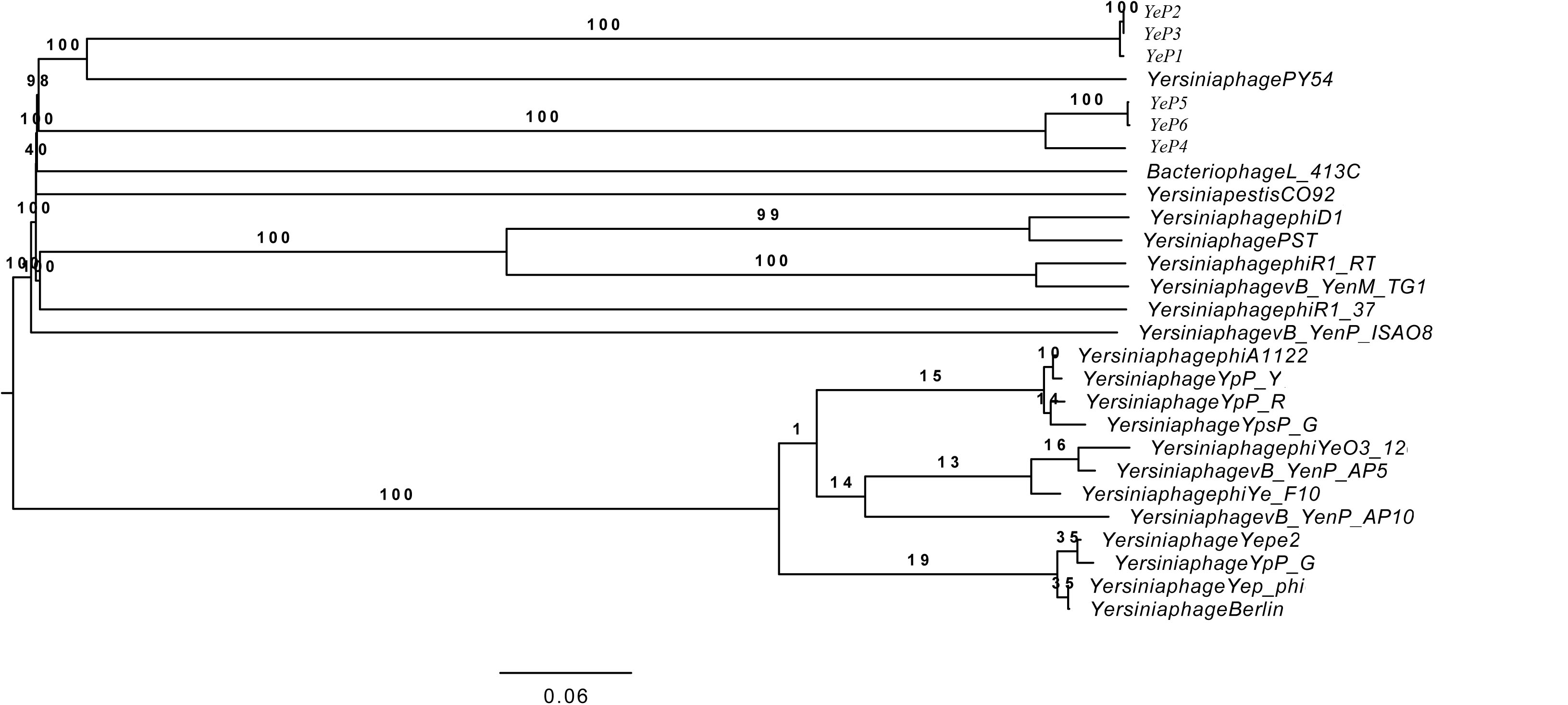

Supplement: FIGURE S2 — The phylogenetic tree of the 27 Yersinia phages genome sequences using the program VICTOR (https://ggdc.dsmz.de/victor.php). [file Image_2.JPEG]
